# Indolium 1 Exerts Activity against Vemurafenib-Resistant Melanoma In Vivo

**DOI:** 10.3390/antiox11050798

**Published:** 2022-04-19

**Authors:** Rakan Radi, Christina Huang, Justin Elsey, Yoon H. Jung, Victor G. Corces, Jack L. Arbiser

**Affiliations:** 1 Department of Dermatology, Emory University School of Medicine, Atlanta, GA 30322, USA; rradi@emory.edu (R.R.); chuan30@emory.edu (C.H.); justin.elsey@emory.edu (J.E.); 2 Department of Human Genetics, Emory University School of Medicine, Atlanta, GA 30322, USA; yoonhee.jung@emory.edu (Y.H.J.); vcorces@emory.edu (V.G.C.); 3 Division of Dermatology, Veterans Affairs Medical Center, Decatur, GA 30033, USA

**Keywords:** melanoma, NADPH oxidase, SOX2, EPHA3, stem cell

## Abstract

The development of targeted therapies (BRAF/MEK inhibitors) and immunotherapy have had a major impact on the treatment of melanoma. However, the majority of patients with advanced melanomas succumb to their disease. The mechanisms of resistance to both targeted therapies and immunotherapies are numerous and have been well-described. These include the alternative activation of BRAF/MEK signaling, novel compensating mutations in additional oncogenes, and loss of neoantigens. There has been limited development of small molecules that target alternative pathways in melanoma in the last two decades. We have previously identified triphenylmethanes as a class that shows activity against a wide variety of tumors. We have synthesized a novel triphenylmethane, indolium 1, and demonstrated its efficacy against an aggressive vemurafenib-resistant melanoma in vivo. Indolium 1 has a novel mechanism of action against melanoma, in that it results in induction of the tumor-suppressor EPHA3. We believe that pre-IND studies are warranted for this novel compound, given its mechanism of action and ability to inhibit the growth of vemurafenib resistant melanoma in vivo.

## 1. Introduction

Melanoma remains the major cause of death due to a skin cancer, and the incidence and number of deaths are increasing. In the last two decades, there has been a major change in the treatment of patients with melanoma, based on the melanoma’s genetics and immunology. Approximately 50–60% of cutaneous melanomas demonstrate the expression of mutant BRAF, and have been targeted by BRAF, and MEK inhibitors [1]. Resistance to these inhibitors has developed through a wide variety of mechanisms, with both intrinsic resistance in existing tumors and the development of novel mutations (extrinsic resistance), which are well-described [2,3]. Other subsets of melanoma benefit less from targeted therapies (uveal melanoma, acral melanoma, melanomas mutant in NRAS and triple-negative melanomas). 

Immunotherapies have also played a major role in the treatment of patients with melanoma. In some instances, long-term remission has been achieved. However, mixed response and relapse is a more common phenomenon. The modes of resistance to immunotherapy have also been well-described. Tumors which are responsive to immunotherapy generally have a high mutational burden, which is likely a source of neoantigens. They also express PDL1, PD1, and CTLA4, although an immunohistochemical test that predicts response to immunotherapy has proven elusive. Finally, both intrinsic resistance due to tumor heterogeneity and extrinsic resistance due to loss of neoantigens or dedifferentiation have been observed. 

Prior studies have shown that additional pathways are activated in cutaneous melanoma, but, surprisingly, little effort has been made to target these pathways in the clinic. We and others have demonstrated that high levels of NADPH oxidase (NOX) are present in aggressive melanomas and that this is accompanied by the activation of AKT [4]. We have also demonstrated that the triphenylmethane, gentian violet, which inhibits NOX, is beneficial in the treatment of cutaneous melanoma in humans [5]. NADPH oxidase is implicated in melanoma progression, as it has been shown to generate reactive oxygen species (ROS) which could, in turn, drive melanoma growth [6]. Inhibiting NOX can also inhibit melanoma cell migration [7]. Thus, NOX inhibition can be a key target for antioxidants [8]. However, gentian violet is not available for systemic use in humans. To address this unmet need, we synthesized a novel triphenylmethane, indolium 1, which has potent in vivo activity against LM36R (the counterpart of vemurafenib-sensitive LM36), which is an aggressive BRAF mutant melanoma that is vemurafenib-resistant [9,10,11]. This cell line was chosen for this study as it is a well-established model of vemurafenib resistance due to elevated reactive oxygen. Of note, LM36R completely lacks expression of the antioxidant enzyme MnSOD, resulting in increased tumor aggressiveness [10]. Targeting vemurafenib-resistant cells in melanoma is of great interest, as, despite the clinical efficacy of selective BRAF inhibitors such as vemurafenib, resistance to treatment often develops [12]. As such, novel means of bypassing vemurafenib resistance could improve care for melanoma patients. Indolium 1 is well-tolerated in mice and, given its efficacy against vemurafenib-resistant melanoma, works through a mechanism that is independent of BRAF. In addition, as a triphenylmethane, it likely has activity as a NOX inhibitor, and appears to downregulate SOX2, a trend seen in other NOX inhibitors including gentian violet [9,13]. ATAC-seq reveals that it induces EPHA3, which is a tumor neoantigen and tumor-suppressor gene, thus demonstrating a novel mechanism of action [14,15,16]. Further development of indolium 1 as a novel therapeutic for human melanoma is warranted.

## 2. Materials and Methods

### 2.1. Synthesis of Indolium 1

**Figure 1:**
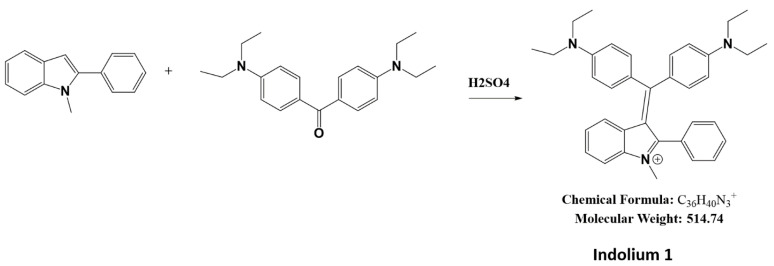
Synthesis of Indolium 1.

A total of 1 g of ethyl Michlers ketone (CAS: 90-93-7, Product # B1275, TCI, Portland, OR, USA) and 0.68 g of 1-methyl-2-phenylindole (CAS: 3558-24-5, Product # 404888, Sigma-Aldrich, St. Louis, MO, USA) were heated with 0.2 mL sulfuric acid at 75 °C until the mixture turned blue (Figure 1). A total of 10 mL of methanol was added, and heated briefly to 75 °C. After 1 h, methanol was added to a total volume of 50 mL, was neutralized with 0.2 g of sodium carbonate, and excess Michlers ketone was extracted with hexane until the hexane layer was no longer yellow. The reaction was then purified by liquid extraction and column chromatography. Purity and structure were assessed by thin-layer chromatography (TLC), liquid chromatography-mass spectrometry (LC-MS) (Figures S1 and S2), and nuclear magnetic resonance (NMR) (Figure S3) at Emory University.

### 2.2. Cell Culture

Roswell Park Memorial Institute-1640 (RPMI-1640) cell medium, trypsin-EDTA, and Dulbecco’s phosphate-buffered saline (DPBS) were purchased from Sigma-Aldrich.

LM36R was maintained in RPMI-1640 supplemented with 10% fetal bovine serum (Atlanta Biologicals, Atlanta, GA, USA), 4 mM glutamine, 100 µg/mL penicillin and 100 µg/mL streptomycin (Sigma-Aldrich) in an atmosphere of 37 °C with 5% CO_2_. Media were changed every 2 days.

### 2.3. Proliferation Assay

A cell proliferation assay was used to study the effect of indolium 1 in vitro. In a 96-well plate, 50,000 LM36 cells were incubated in 6 lanes (4 wells in each lane) with 200 μL/well of the aforementioned growth media. After 24 h, the media was changed to treated media. Lane 1 served as a control, with added DMSO. Lane 2 was treated with indolium 1 (1 μM) (stock solution: 5.14 mg/mL dissolved in DMSO). Lanes 3 and 4 were treated with 10 μM MEK inhibitor U0126 (stock solution: 1 mg/mL in DMSO) (Promega Corporation, Madison, WI, USA) and 10 μM Calbiochem Akt Inhibitor II (Product # 124008, Sigma-Aldrich) (stock solution: 1 mg/mL in DMSO). Lane 5 was treated with a combination of indolium 1 (1 μM) and MEK inhibitor U0126 (10 μM), while Lane 6 was treated with a combination of indolium 1 (1 μM) and Akt inhibitor II (10 μM). Cells were incubated for 24 h. A total of 20 μL of alamarBlue™ cell viability reagent (Thermo Fischer Scientific, Waltham, MA, USA) was added to each well. After 4 h, the plate was read, following manufacturer’s instructions (SpectraMax 340PC384, Molecular Devices, San Jose, CA, USA) and the data were processed using Softmax Pro software (Molecular Devices) and Microsoft Office Excel (Microsoft Corporation, Redmond, WA, USA). An unpaired, two-tailed Student’s *t*-test was performed to determine the significant difference between the control and each treatment group, along with between the indolium 1 only versus indolium 1 plus MEK/AKT inhibitor, with significance determined at *p* < 0.05. 

### 2.4. Western Blotting

LM36R cells were grown in six T75 flasks. Upon reaching 70% confluence, cell media was replaced with media with concentrations of 0, 0.1, 0.5, 1, 2.5, and 5 μM Indolium 1. After 24 h, cells were lysed in Pierce RIPA buffer (25 mM Tris-HCl, 150 mM NaCl, 1% NP-40, 1% sodium deoxycholate, 0.1% sodium dodecyl sulfate, pH 7.6) (Thermo Fisher Scientific, Waltham, MA, USA) supplemented with HALT protease phosphatase inhibitor cocktails (Thermo Fischer Scientific) and 0.5 M EDTA solution (Thermo Fischer Scientific). Cell lysates were incubated on ice for 30 min, and then centrifuged at 16,000× *g* at 4 °C for 20 min. Protein concentration was determined using the Pierce BCA Protein Assay Kit (Thermo Fischer Scientific) and then normalized. NuPage LDS Sample Buffer (4×) and beta-mercaptoethanol (Sigma-Aldrich) were added to the samples, which were subsequently boiled. A total of 40 µg of sample protein were loaded into each well of NuPAGE 4–12% Bis-Tris precast gels (Thermo Fischer Scientific) in MOPS buffer (Thermo Fisher Scientific) against Precision Plus Protein Dual Color Standards (Bio-Rad Laboratories, Hercules, CA, USA). Proteins were transferred onto polyvinylidene difluoride membrane using Transblot Turbo system (Bio-Rad Laboratories). Membranes were blocked for 1 h at room temperature in 5% non-fat dry milk in 0.1% Tween-Tris-Buffered Saline and probed with Rb (#PA5-27215, Thermo Fischer Scientific) pMAPK (9101S, Cell Signaling Technology, Danvers, MA, USA), pAkt (#4060S, Cell Signaling Technology), SOX2 (ab171380, Abcam, Cambridge, UK) and beta-tubulin (#2146S, Cell Signaling Technology) antibodies at 1:1000 dilutions in 5% bovine serum albumin at 4 °C overnight. The membranes were then incubated in HRP-linked anti-rabbit IgG secondary antibody (#7074S, Cell Signaling Technologies) or anti-mouse IgG secondary antibody (#7076S, Cell Signaling Technologies) with a 1:1000 dilution for 1 h at room temperature. Antibody signal was detected using SuperSignal West Pico chemiluminescence substrate (Thermo Fisher Scientific) with Medical X-Ray Film (Super Rx-N, Fujifilm, Minato City, Japan) and digitally scanned (Lanier LD645C, Ricoh Electronics, Lawrenceville, GA, USA). 

### 2.5. In Vivo LM36R Xenograft Model

The xenograft model was developed and approved by the Institutional Animal Care and Use Committee (IACUC) of Emory University, and all methods were performed in accordance with the approved IACUC protocol guidelines and regulations. LM36R cell suspension in growth medium was inoculated at 1 × 10^6^ cells/mouse in the right flank of athymic Nu/Nu nude male mice (*n* = 5 per group) purchased from the Charles River Laboratories (Wilmington, MA, USA). Indolium 1 was prepared by dissolving 3.75 mg into 1 mL of ethanol 7. A total of 100 μL of this solution was then diluted to the final concentration in 1 mL of 20% soy-fat Intralipid (Frensenius Kabi, Bad Homburg, Germany) prior to each injection and vortexed vigorously. Vehicle control (ethanol in Intralipid) or indolium 1 was administered intraperitoneally three times a week at 3 mg/kg/dose for four weeks. Indolium 1 treatment was initiated on the second day after the tumor cell injection, and the tumor volume, as well as the weight of the animals, were recorded weekly thereafter. Animals were sacrificed and tumors were harvested after four weeks of treatment, when tumor volumes reached experimental endpoint, as determined by IACUC protocol.

### 2.6. Immunohistochemistry of In Vivo LM36R Tumor Model

Tumor samples were resected and embedded in formalin, processed, and stained at Winship Cancer Institute Research Histology Core laboratory (Atlanta, GA, USA). 

Formalin-fixed and paraffin-embedded tissue sections from each group were cut to a 5-μm thickness and air-dried. Processing was performed using Ventana DISCOVERY Ultra automated immunohistochemistry stainer (Ventana Medical Systems, Tuscon, AZ, USA). Slides were deparaffinized with EZ-Prep (# 05279771001, Ventana) and then were antigen retrieved for 64 min with CC1 reagent (#950-500, Ventana). Rabbit anti-Eph receptor A3 (EphA3) antibody (ab126261) (Abcam) diluted at 1:50, Rabbit anti-Nkx3.2 (#PA5-21108 diluted at 1:500, Thermo Fischer Scientific) and mouse anti-Rb1 antibody (554136, BD Biosciences, Franklin Lakes, NJ, USA) diluted at 1:100 was applied and incubated for 40 min. For the first two stains, DISCOVERY OmniMap anti-Rabbit HRP (Roche, Basel, Switzerland) was applied and incubated for 12 min, and the detection was completed in combination with DISCOVERY ChromoMap DAB kit (Roche) for anti-EphA3 antibody and DISCOVERY Red kit (Roche) for anti-Nkx3.2 antibody, as per manufacturer recommendations. For the Rb1 stain, DISCOVERY OmniMap anti-mouse HRP (Roche) was applied and incubated for 12 min and the detection was completed in combination with DISCOVERY ChromoMap DAB kit (Roche). Slides were counterstained with hematoxylin for 8 min. Slides were then dehydrated, cover-slipped, and evaluated by light microscopy. Whole imaging was performed on the Hamatsu Nanozoomer HT 2.0 (Hamamatsu Photonics, Hamamatsu City, Japan). 

### 2.7. Assay for Transposase-Accessible Chromatin Using Sequencing (ATAC-seq)

ATAC-seq was carried out using the Omni-ATAC protocol [17,18]. Nuclei were isolated from each tumor sample via Dounce homogenization and density centrifugation. After nuclei were counted, the 50,000 nuclei pellet was resuspended in the transposase reaction mix (25 μL 2× TD buffer, 2.5 μL transposase, 16.5 μL PBS, 0.5 μL 1% digitonin, 0.5 μL 10% Tween-20, 5 μL H_2_O) and incubated for 30 min at 37 °C in a thermoshaker with 600 RPM constant shaking. Following incubation, DNA was isolated with Zymo DNA Clean and Concentrator kit. Library amplification was carried out with 2× KAPA HiFi mix (Kapa Biosystems, Wilmington, MA, USA) and 1.25 µM indexed primers using the following PCR conditions: 72 °C for 5 min; 98 °C for 30 s; and 10–11 cycles at 98 °C for 10 s, 63 °C for 30 s, and 72 °C for 1 min.

### 2.8. Analysis of ATAC-seq Data

All libraries were sequenced using Illumina Novaseq 6000 sequencers and 50 bp paired-end format. Paired reads were aligned to the human reference genome hg38 using Bowtie2. ATAC-seq reads were aligned using default parameters, except -X 2000 -m 1. PCR duplicates were removed using Picard Tools (http://picard.sourceforge.net; https://broadinstitute.github.io/picard/ accessed on 8 August 2021). To adjust for fragment size, we aligned all reads as + strands offset by +4 bp and—strands offset by −5 bp. For all ATAC-seq datasets, subnucleosome size and mono-nucleosome-size reads were separated by choosing fragments of 50–115 bp and 180–247 bp in length, respectively. MACS2 was used for peak calling of subnucleosomal reads, which represent bound transcription factors. Differentially accessible sites were identified using the R package “Diffbind”, with a cut-off *p*-value ≤ 0.05 and fold change ≥ 2.

### 2.9. Statistical Analysis

The statistical analysis of tumor volumes was performed as previously described, and statistical analyses were performed using the Microsoft Office Excel (Microsoft Corporation) and GraphPad Prism software (GraphPad Software, La Jolla, CA, USA). In brief, tumor volume was calculated using the formula, volume = (L × W2) × 0.52, where L was defined to be the longer dimension of the tumor. Replicate size per group was 5, and unpaired two-tailed Student’s *t*-test was performed to determine the significant difference between the two groups, with significance determined at *p* < 0.05.

## 3. Results

**Figure 2:**
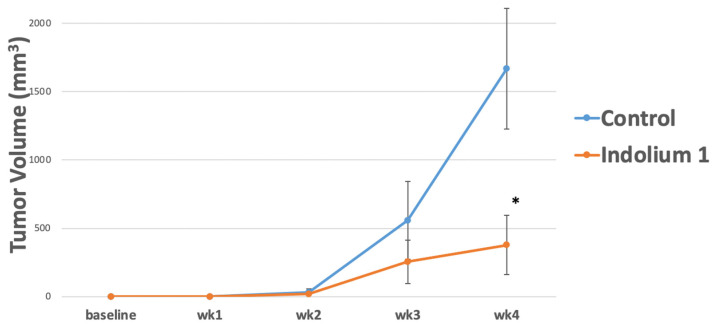
Tumor Growth in control versus Indolium 1-treated mice. Indolium 1 treatment results in reduced tumor volume over 4 weeks. Mice were inoculated with LM36R, a vemurafenib-resistant melanoma cell line, at 1 × 10^6^ cells/mouse. Mice were then treated with Indolium via IP administration three times a week at a concentration of 3 mg/kg/dose. Tumor growth was compared to control vehicle (sterile water). Results indicate a possible reduction in tumor growth over the period of observance. (N = 5/group, *p*-value = 0.03) at week 4. * = Statistically significant reduction in tumor volume compared to control (*p* < 0.05).

In order to investigate indolium 1 activity against LM36R, vemurafenib-resistant melanoma cell line, in vivo, 10 mice were inoculated at 1 × 10^6^ cells/mouse. Mice were then treated with indolium 1 via IP administration three times a week at a concentration of 3 mg/kg/week. The results suggest a significant reduction in tumor volume in indolium 1-treated mice compared to the control (Figure 2). 

**Figure 3:**
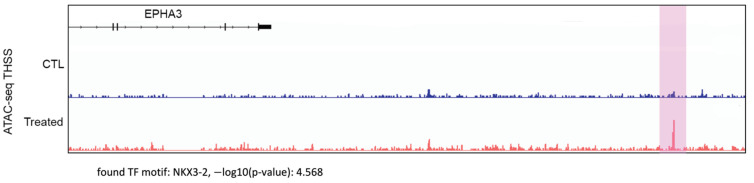
ATAC-seq analysis comparing chromatin accessibility in control mouse tumor tissue (“CTL”, top, in blue) versus Indolium 1-treated mouse tumor tissue (“Treated”, bottom, in red) LM36R cells in the *EPHA3* gene. The highlighted region shows a statistically significant (*p*-value = 2.7 × 10^−5^) difference in chromatin accessibility for the region corresponding to the NKX3-2 binding motif in the *EPHA3* gene.

To gain insights into the mechanisms by which indolium 1 caused a decrease in LM36R tumor volume, we analyzed possible changes in chromatin structure in the tumor cells using ATAC-seq. *EPHA3* was one of the most upregulated genes in terms of chromatin accessibility (Figure 3). One region of the *EPHA3* gene that had significantly higher chromatin accessibility (highlighted in purple) contains the binding motif for the NKX3-2 transcription factor.

**Figure 4:**
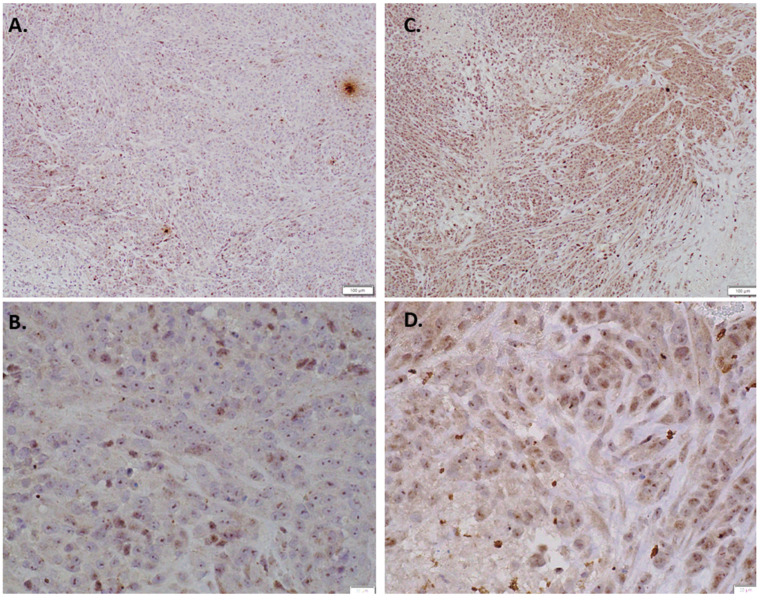
Immunohistochemical staining EPH receptor A3 staining with (**A**) Control (10×) (**B**) Control (40×) (**C**) Indolium 1 Treatment (10×) (**D**) Indolium 1 Treatment (40×). Note increased EPHA3 staining in treated tumors (**C**,**D**).

**Figure 5:**
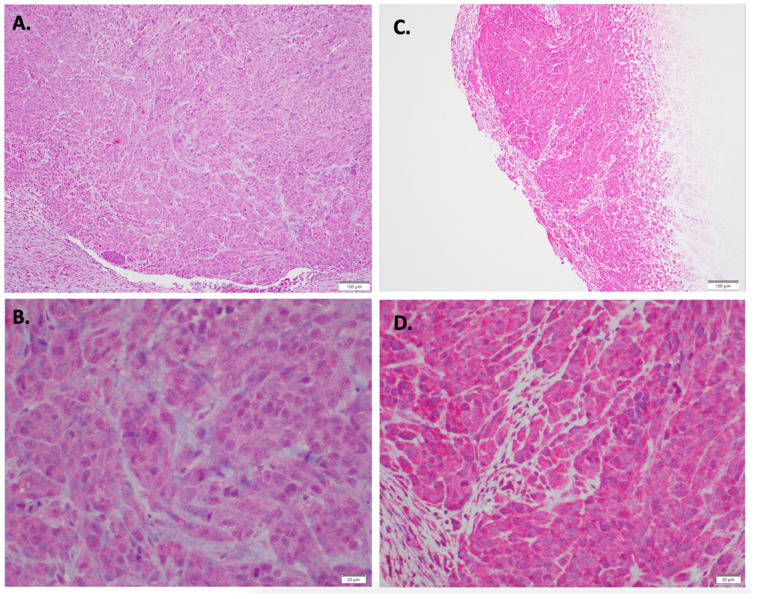
Immunohistochemical staining Nkx3.2 staining with (**A**) Control (10×) (**B**) Control (40×) (**C**) Indolium 1 Treatment (10×) (**D**) Indolium 1 Treatment (40×). Note increased Nkx3.2 staining in treated tumors (**C**,**D**).

Immunohistochemical staining results were consistent with this finding, as indolium 1-treated tumor tissue samples had more positivity for EPHA3 compared to the controls (Figure 4). Furthermore, immunohistochemical staining for Nkx3.2 also showed upregulated Nkx3.2 expression in indolium-treated cells compared to control cells (Figure 5). 

**Figure 6:**
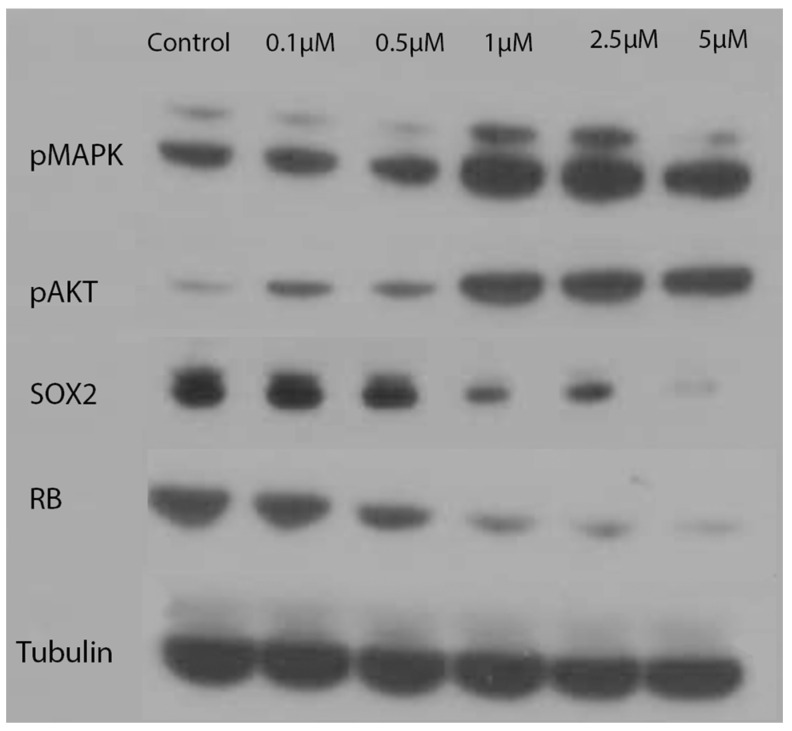
Western Blotting: Levels of pMAPK, pAKT, SOX2, RB, and Tubulin with concentrations of indolium 1 treatment. Tubulin serves as a loading control. LM36R cells were treated with a dose response for 24 h.

To further explore the effects that indolium 1 has on cells, which could have led to a reduction in tumor size, LM36R cells were treated with varying concentrations of indolium 1. Subsequently, it was found that pMAPK and pAKT levels tended to increase with indolium 1 treatment, while levels of RB decreased with increasing concentrations of indolium 1 treatment. Furthermore, decreasing levels of SOX2 with increasing concentrations of indolium 1 treatment suggests that indolium 1 has NOX inhibitory activity similar to that of gentian violet, a known NOX inhibitor (as SOX2 expression is similarly reduced by gentian violet treatment) [9]. Beta-tubulin was used to confirm even protein concentrations in each sample (Figure 6). 

**Figure 7:**
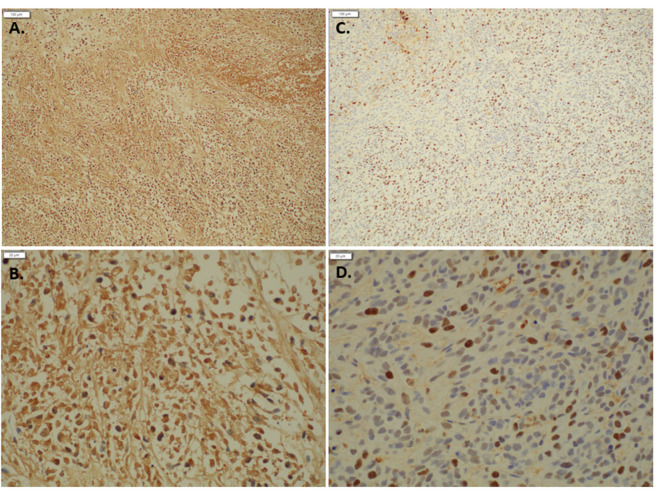
Immunohistochemical staining Retinoblastoma 1 staining with (**A**) Control (10×) (**B**) Control (40×) (**C**) Indolium 1 Treatment (10×) (**D**) Indolium 1 Treatment (40×). Note decreased RB staining in treated tumors (**C**,**D**).

Immunohistochemical staining for RB showed consistent results with the Western blot, with indolium 1-treated tumor tissue expressing less RB compared with control tissue (Figure 7). Thus, the reduction in RB staining can serve as a biomarker of Indolium 1 activity.

**Figure 8:**
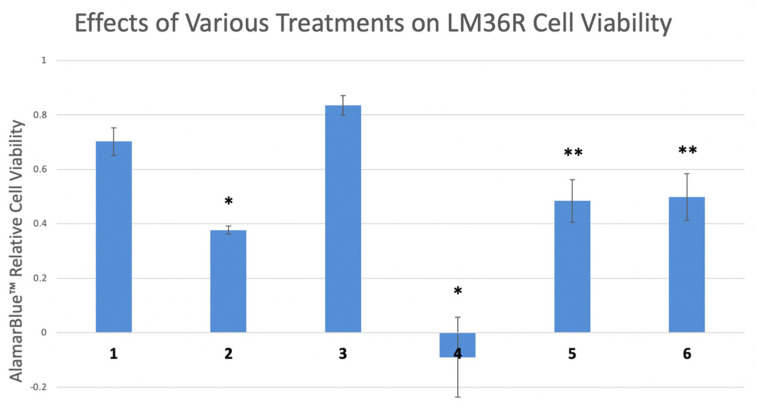
Relative cell viability of LM36R cells treated with certain compounds. Lanes: (1) Control (2) Indolium 1 (1 μM) (3) MEK Inhibitor U0126 (10 μM) (4) Akt Inhibitor II (10 μM) (5) Indolium 1 (1 μM) + MEK Inhibitor U0126 (10 μM) (6) Indolium 1 + Akt Inhibitor II (10 μM). * = Statistically significant lower cell viability compared to control (*p* < 0.05). ** = Statistically significant lower cell viability compared to control and statistically higher cell viability than Indolium 1 treatment (lane 2) alone. Inhibition of MAPK and AKT reverses cell death caused by Indolium 1.

Furthermore, the induction of MAPK and AKT seem to contribute to reduction in cell viability in LM36R cells treated with indolium 1. In a cell viability assay, cells were treated with indolium 1, an MEK inhibitor (inhibiting the MAPK-signaling pathway directly upstream of MAPK), and AKT inhibitor. Cells were also treated with a combination of indolium 1 and each inhibitor. Co-treatment with the MEK inhibitor inhibited cell death, as cell viability was significantly higher in the indolium 1 and MEK-inhibitor group compared to indolium 1 alone. The same effect was observed when cells were co-treated with AKT inhibitor and indolium 1 (Figure 8). 

## 4. Discussion

The treatment of advanced melanoma was greatly impacted by targeted therapies and immunotherapies. However, the vast majority of patients with advanced melanoma succumb to their disease, due to resistance to these agents.

We have synthesized and evaluated a novel small molecule, indolium 1, which has activity against vemurafenib-resistant BRAF mutant melanoma. We analyzed vehicle versus indolium-treated tumors in mice for potential biomarkers and for an insight into the mechanism of indolium 1 in vivo. 

One of the most upregulated genes in terms of chromatin accessibility is EPHA3, which has relevance in melanoma biology [19]. EPHA3 is a receptor tyrosine kinase that binds ligands ephrinA5 and IL-26 [20,21]. While tumor stimulatory and inhibitory functions have been ascribed to EPHA3, most evidence suggest that EPHA3 is a tumor suppressor. First, inactivating mutations in EPHA3 are often found in high-grade malignancies [22,23]. Second, the activation of wild-type EPHA3 causes downregulation of AKT, which has previously been implicated in melanoma progression [20]. Third, EPHA3 has been shown to be a tumor neoantigen in melanoma [24]. Finally, a novel antibody-based therapeutic directed against EPHA3, and therapies that induce EPHA3 could sensitize melanoma to antibodies to EPHA3 [25,26].

Surprisingly, indolium 1 causes loss of expression of the pRB tumor-suppressor gene. This might be seen as a negative for an antitumor agent, but more recent studies demonstrate that there are oncogenic functions of pRB. Cells lacking pRB, but not RB homologs p107 and p130, have greatly increased resistance to transformation with oncogenic RAS, compared to isogenic wild-type fibroblasts [27]. The complete loss of pRB is mostly confined to retinoblastoma, small-cell carcinoma of the lung, and osteosarcoma. In contrast, a high-level expression of pRB is associated with a poor prognosis in melanoma [28]. Further support for an oncogenic role of pRB is provided by the study of Chicas et al., in which loss of pRB causes aberrant proliferation and defective cell-cycle exit, leading to cell death [29]. The loss of pRB expression is noted on both Western blot analysis and by immunohistochemistry of treated tumors. This provides a biomarker activity for indolium 1.

The treatment of LM36R melanoma cells also leads to a surprising induction of phosphorylated MAP kinase and AKT. This could be a protective response to indolium 1 expression, or a requirement for indolium 1-induced cell death. In order to determine these possibilities, we treated LM36R cells in the presence of inhibitors of MAPK, and AKT to determine whether these inhibitors would protect or potentiate cell death due to indolium 1. Both inhibitors provided protection against indolium 1-induced cell death. This has important potential clinical implications. First, resistance to targeted therapies is associated with the re-expression of MAP kinase and AKT, so indolium 1 would be well-positioned to treat melanoma that has recurred after targeted therapies. Second, one would not want to concurrently use BRAF/MEK inhibitors with indolium 1, but could perhaps use them in a sequential fashion, as concurrent use might blunt the efficacy of indolium 1. Finally, indolium 1 may cause increased differentiation, which may also lead to the development of additional neoantigens [30].

The triphenylmethane class of small molecules has been shown to exhibit antioxidant activity in glioblastoma and medulloblastoma (imipramine blue) and melanoma (gentian violet) [31]. Inhibition of NADPH oxidase by gentian violet has shown to downregulate SOX2 as the major target. SOX2 is one of the Yamanaka cell factors and is a poor prognostic factor in melanoma [32,33]. We demonstrate that indolium 1 downregulates SOX2 in a dose dependent fashion. Of further importance, tumors that have lost pRB upregulate SOX2 as consequence [34]. An agent that downregulates both pRB and SOX2 would be expected to have potent activity against melanoma.

## 5. Conclusions

The current landscape of therapies for advanced melanoma consists primarily of BRAF/MEK inhibitors to target BRAF mutant melanoma, and immunotherapies to target all comers in advanced melanoma. Unfortunately, resistance to all these therapies is the rule. Indolium1 is a molecule with a novel mechanism of action, which targets, rather than inhibits, MAPK and AKT activation. Further clinical development of this first-in-class molecule for melanoma is warranted.

## 6. Patents

Emory University is filing a patent on indolium 1.

## Data Availability

ATAC-seq data are available from NCBI’s Gene Expression Omnibus (GEO) under accession number GSE200882.

## Supplementary Materials

The following supporting information can be downloaded at: https://www.mdpi.com/article/10.3390/antiox11050798/s1, Figure S1: Liquid chromatography data for indolium 1; Figure S2: Mass spectrometry data for indolium 1; Figure S3: HNMR profile for indolium 1.
