# Supplementary material for: Indolium 1 Exerts Activity against Vemurafenib-Resistant Melanoma In Vivo
Source: Antioxidants (Basel). 2022 Apr 19;11(5):798. doi: 10.3390/antiox11050798 (PMC9137681; doi:10.3390/antiox11050798)
Supplement: Supplementary file 1 [file antioxidants-11-00798-s001.zip › antioxidants-1646465-supplementary.pdf]

Supplemental Figures:

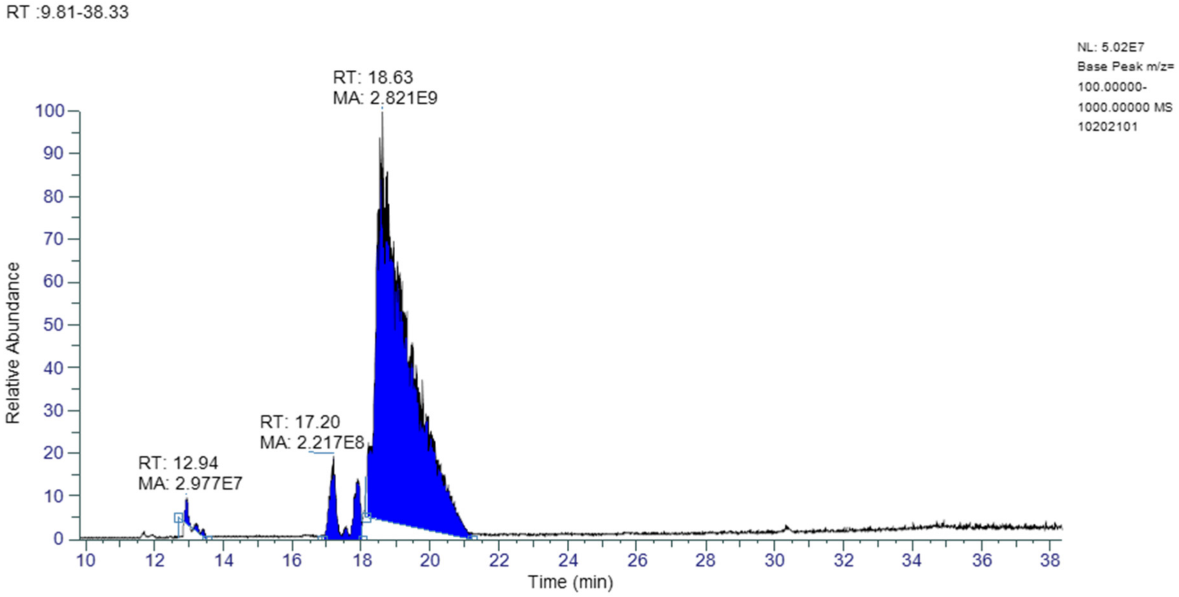

RT (Min)	Peak Area	% Area	% Height	Integration Method
12.94405	29767039	0.97	4.96	Manual
17.20415	2.22E+08	7.22	16.09	Manual
18.62597	2.82E+09	91.81	78.95	Manual

Supplemental Figure S1. Liquid Chromatography data for Indolium 1.

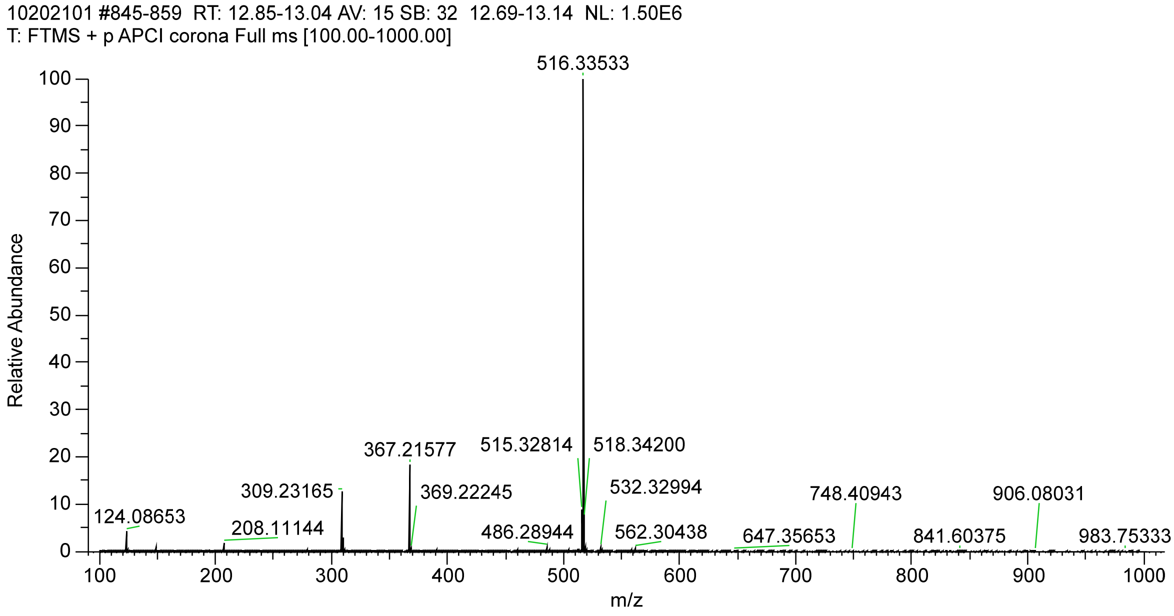

Supplemental Figure S2. Mass Spectrometry data for Indolium 1.

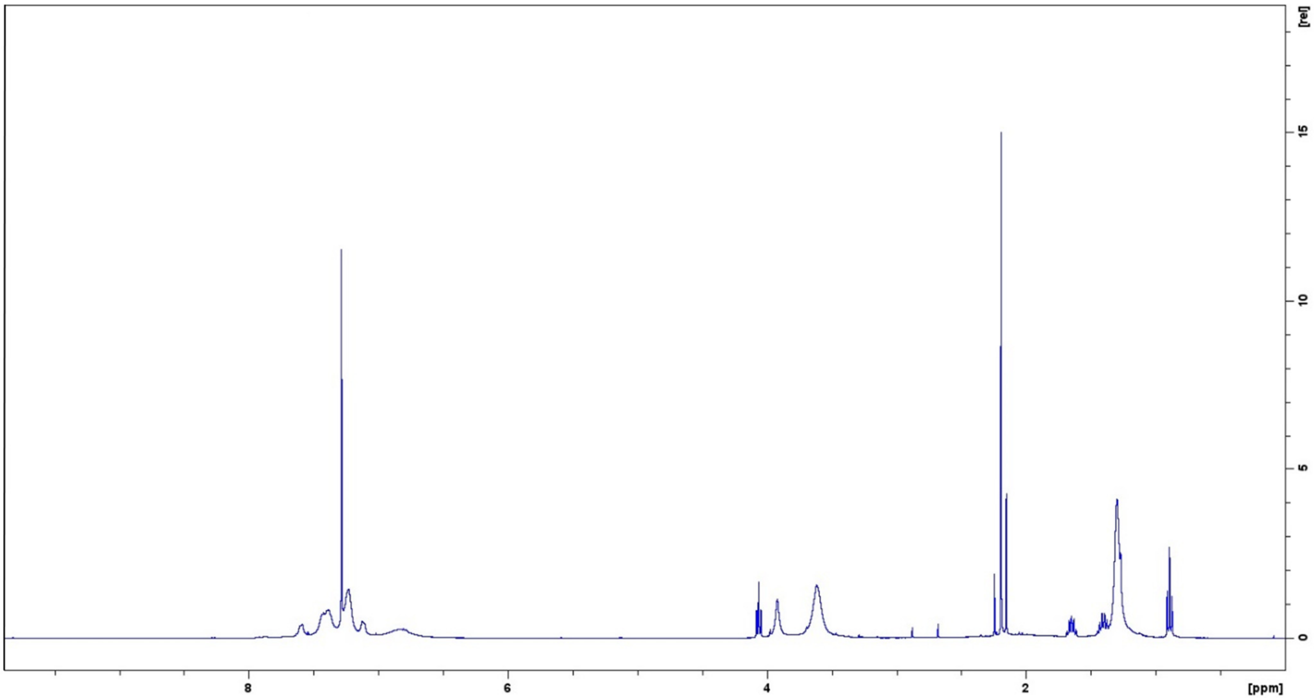

Supplemental Figure S3. HNMR Profile for Indolium 1.
